# Draft Genome Sequence of *Nitrosospira* sp. Strain APG3, a Psychrotolerant Ammonia-Oxidizing Bacterium Isolated from Sandy Lake Sediment

**DOI:** 10.1128/genomeA.00930-13

**Published:** 2013-11-07

**Authors:** Juan C. Garcia, Hidetoshi Urakawa, Vang Q. Le, Lisa Y. Stein, Martin G. Klotz, Jeppe L. Nielsen

**Affiliations:** Department of Marine and Ecological Sciences, Florida Gulf Coast University, Fort Myers, Florida, USAa; Department of Biotechnology, Chemistry and Environmental Engineering, Aalborg University, Aalborg, Denmarkb; Department of Biological Sciences, University of Alberta, Edmonton, Alberta, Canadac; Department of Biology, University of North Carolina, Charlotte, North Carolina, USAd

## Abstract

Bacteria in the genus *Nitrosospira* play vital roles in the nitrogen cycle. *Nitrosospira* sp. strain APG3 is a psychrotolerant betaproteobacterial ammonia-oxidizing bacterium isolated from freshwater lake sediment. The draft genome revealed that it represents a new species of cluster 0 *Nitrosospira*, which is presently not represented by described species.

## GENOME ANNOUNCEMENT

Bacteria in the genera *Nitrosospira* and *Nitrosomonas*, family *Nitrosomonadaceae*, class *Betaproteobacteria*, are obligate aerobic chemolithotrophic ammonia-oxidizing bacteria (AOB) that facilitate nitritation, the first step of nitrification. As determined based on 16S rRNA gene phylogeny, the genus *Nitrosospira* is represented by 5 clusters (lineages). Three previously described species, *Nitrosospira briensis* (type species), *N. tenuis*, and *N. multiformis*, belong to cluster 3 (1, 2), whereas the other clusters are not represented by described species in culture. To date the only publically available *Nitrosospira* genome is that of *N. multiformis* ATCC 25196^T^ (3).

*Nitrosospira* sp. strain APG3 was isolated from sandy freshwater lake sediment in Seattle, WA. 16S rRNA sequence analysis revealed that APG3 belongs to *Nitrosospira* cluster 0, which is presently not represented by described species. APG3 grows at 4°C but does not grow at 35°C, indicating that this bacterium is psychrotolerant. APG3 is also able to grow at pH 5, which is the lowest pH reported for AOB in culture (4, 5).

The draft genome of APG3 was sequenced using the Illumina HiSeq 2000 platform (San Diego, CA) with 2 × 150 bp and a 50-bp overlap using a paired-end library (1,310,942,146 reads). The reads generated by the Illumina HiSeq 2000 were assembled using CLC Genomics Workbench v 5.0 (CLC bio), and the resulting contigs were curated by CodonCode Aligner v 3.7 (CodonCode Corp.). The assembled contigs were analyzed in the Rapid Annotation using Subsystem Technology (RAST) annotation server for subsystem classification and functional annotation (6). Additional genome predictions, annotations, and checks for compliance with EMBL recommendations were performed by use of the EMBL validator software and by a curation team.

The draft genome sequence comprises 3,107,181 bases at 272-fold coverage. The assembled draft genome consists of 84 contigs with an average size of 41,181 bp and a G+C content of 53.6%. The draft genome contains 3,147 protein-coding DNA sequences, 44 tRNA genes, and a single 16S-23S-5S rRNA operon. The closest neighbor of APG3 was identified as *N. multiformis* ATCC 25196^T^. The average nucleotide identity (ANI) (7, 8) calculated between APG3 and *N. multiformis* ATCC 25196^T^ was 75.45%, which was significantly lower than the accepted cutoff at the species level.

We identified genes encoding an inventory of proteins implicated in ammonia oxidation by AOB, including ammonia-monooxygenase (EC 1.14.99.39), hydroxylamine dehydrogenase (EC 1.7.2.6), and cytochromes c554 and cM552 as well as nitrosocyanin (9). As in *N. multiformis* ATCC 25196^T^, a gene cluster encoding NAD-reducing hydrogen dehydrogenase (EC 1.12.1.2) was present in the APG3 draft genome, which supports the potential of nitrosospiras to utilize hydrogen as an alternative energy source (3). Many but not all AOB also use urea for chemolithotrophic growth (3, 10); APG3 is able to use urea as an alternate source for energy reductant and carbon. The finding of a urea hydrolase operon supports this physiological observation. The APG3 genome also included genes encoding an inventory implicated in the assimilation of N, S, P, and C (the Calvin-Benson-Bassham cycle) in *N. multiformis* (3). Genes encoding incomplete denitrification inventories, such as nitrite reductase (*nirK*) and nitric oxide reductases, were also present. Like *N. multiformis*, the APG3 genome encodes a large number of chemotaxis- and flagellum-associated proteins (3). Further genome-closing, annotation and genome comparisons with other AOB will provide additional insights into the unique evolution and ecological adaptation of this microorganism.

### Nucleotide sequence accession numbers.

This whole-genome shotgun project has been deposited in DDBJ/EMBL/GenBank under the accession number CAUA00000000. The version described in this paper is the first version, CAUA01000000.
